# Changes in body mass index and behavioral health among adolescents in military families during the COVID-19 pandemic: a retrospective cohort study

**DOI:** 10.1186/s12889-023-16548-0

**Published:** 2023-08-24

**Authors:** Tracey Pérez Koehlmoos, Cathaleen Madsen, Amanda Banaag, Terry Adirim

**Affiliations:** 1 Department of Preventive Medicine and Biostatistics, Uniformed Services University of the Health Sciences, 4301 Jones Bridge Road, Bethesda, MD 20814 USA; 2 Center for Health Services Research, 6720A Rockledge Drive, Suite 250, Bethesda, MD 20817 USA; 3 The Henry M. Jackson Foundation for the Advancement of Military Medicine, Inc, 6720A Rockledge Drive, Bethesda, MD 20817 USA; 4 Department of Pediatrics, Uniformed Services University of the Health Sciences, 4301 Jones Bridge Road, Bethesda, MD 20814 USA

**Keywords:** Adolescent Health, Adolescent Behavioral Health, COVID-19, Large Datasets, Electronic Health Records

## Abstract

**Background:**

Widely published findings from the COVID-19 pandemic show adverse effects on body mass index (BMI) and behavioral health in both adults and children, due to factors such as illness, job loss, and limited opportunity for physical and social activity. This study investigated whether these adverse effects were mitigated in adolescents from military families, who are universally insured with consistent access to healthcare, and who generally have at least one parent who must adhere to physical and mental fitness as a condition of employment.

**Methods:**

We conducted a cohort study using two groups of adolescents receiving care in the U.S. Military Health System during the COVID-19 pandemic; one for changes in Body Mass Index (BMI) and the second for changes in behavioral health diagnoses, using TRICARE claims data. Beneficiaries (160,037) ages 13 to 15 years in fiscal years 2017–2018, were followed up during October 2020 to June 2021.

**Results:**

Among the BMI cohort, 44.32% of underweight adolescents moved to healthy weight, 28.48% from overweight to obese, and 3.7% from healthy weight to underweight. Prevalence of behavioral disorders showed an overall 29.01% percent increase during the study period, which included in mood (86.75%) and anxiety (86.49%) disorders, suicide ideation (42.69%), and suicide attempts (77.23%). Decreases in percent change were observed in conduct disorders (-15.93%) and ADD/ADHD (-8.61%).

**Conclusions:**

Adolescents in military families experienced adverse health outcomes during the pandemic at approximately the same rates as those in non-military families, suggesting that universal insurance and military culture were not significantly mitigating factors. Obesity and underweight present significant opportunities to intervene in areas such as exercise and food access. Decreased conduct disorders and ADD/ADHD may reflect lower prevalence due to favorable home environment, or lower rates of diagnosis and referral; however, increased rates of anxiety, mood disorders, suicide ideation and attempt are especially concerning. Care should be taken to ensure that adolescents receive consistent opportunity for physical activity and social interaction, and those at risk for suicide should receive active monitoring and appropriate referral to behavioral healthcare providers.

**Supplementary Information:**

The online version contains supplementary material available at 10.1186/s12889-023-16548-0.

## Background

The COVID-19 pandemic worsened existing high-risk conditions in the adolescent population. Rates of mental and behavioral health disorders in children and adolescents are as high as 20% [1] and overweight and obesity are predicted to approach 50% by 2030 [2]. Current literature confirms that the COVID-19 pandemic has exacerbated these conditions, with 29% of parents reporting worsening mental health of their children and adolescents [3] and published sources describing significant increase in child and adolescent body mass index (BMI) [4]. Loss of in-person schooling, peer social engagement, structured activities, school-provided mental health services, and school-based nutrition programs, are significant contributors in worsening child health during the pandemic [4, 5]. Parental illness and job loss also pose significant emotional and economic stressors with potentially negative impacts to child health, for instance, if parental stress impacts child behavior or reduced finances impact access to food and other necessities.

Children in military families face many of the same challenges as their peers, as they often live in the same neighborhoods and attend the same schools as their counterparts in non-military families. Research from 2017 and 2018 found that rates of obesity and behavioral health conditions in this population were consistent with rates in the greater U.S. child and adolescent population [6]. Taken together, this suggests that children in military families were subject to many of the same disruptions from the COVID-19 pandemic that have caused increased BMI and increased rates of mental health disorders among children in the greater U.S. population. However, military families have several advantages not guaranteed to non-military families. First, they are universally insured, and may receive care at military facilities or from private-sector providers who accept TRICARE insurance. Second, the military parents are generally healthy and are guaranteed employment through the end of their contracts. Third, as they become young adults, children from military families enter military service themselves at a higher rate than do those from non-military families [7], which may provide a protective factor in maintaining BMI and behavioral health at levels suitable to meet recruiting standards [8].

Military children receive health care services through the Military Health System (MHS), whose beneficiaries form a nationally-representative, geographically distributed, socioeconomically diverse population. The healthcare data for approximately 2.3 million military dependent children and young adults are captured in the MHS Data Repository providing an opportunity to examine changes to BMI and behavioral health at a population level. This study investigates these changes among a previously assessed cohort of children and adolescents [6] during the COVID-19 pandemic. This study is expected to inform discussion of universal insurance and military culture as potentially protective factors mitigating pandemic-associated adverse health changes among adolescents and young adults. The inclusion of beneficiaries across racial, gender, and socioeconomic categories provides an additional opportunity to examine equity in this population.

## Methods

### Study design and data source

We extracted TRICARE claims data from the MHS Data Repository (MDR) and developed two adolescent cohorts in fiscal years (FY) 2017 to 2018 (October 1, 2016 to September 30, 2018), with a health status follow-up period in FY 2020 to June 2021 (October 1, 2019 to June 30, 2021). The two cohorts consisted of a BMI cohort (1) and behavioral health cohort (2) and were identified as follows: 1) TRICARE Prime dependent adolescents ages 13 to 15 years in FY 2017 to 2018 with a recorded BMI in both FY 2017 to 2018, and 2) all TRICARE Prime dependent adolescents ages 13 to 15 years in FY 2017 to 2018. The end date period covers care delivered during the COVID-19 pandemic up to the most recent and complete data available at the time of the study (FY 2020 to June 2021).

### Adolescent BMI cohort

Inclusion criteria of the BMI cohort consisted of dependent adolescents of the ages 13 to 15 years in FY 2017 to 2018 with a recorded BMI in both evaluated time periods (FY 2017 to 2018 and FY 2020 to June 2021). Recorded BMI included either a calculated BMI from height and weight measurements and age and sex growth percentiles, or an International Classification of Diseases, 10^th^ revision (ICD-10), diagnostic code indicating the adolescent’s BMI category—for all adolescents under the age of 20 [7]. However, some adolescents in the cohort were 20 or older during the follow-up time point (FY 2020 to June 2021) and therefore BMI calculations and diagnostic coding for adults were utilized. BMI was defined and categorized in accordance with the Centers for Disease Control’s pediatric and adult cutoffs: Underweight (< 5^th^ percentile for age or adult BMI < 18.5), Healthy Weight (5^th^ to less than 85^th^ percentile for age or adult BMI 18.5–24.9), Overweight (85^th^ to less than 95^th^ percentile for age or adult BMI 25.0–29.9), and Obese (≥ 95^th^ percentile for age or adult BMI > 30.0) [9, 10]. If an adolescent had both a calculated and a diagnosed BMI, then the calculated BMI was used in analysis; and the most recent BMI recording in both time periods was used in analyses if multiple were found during each time point.

### Adolescent behavioral health

All dependent adolescents of the ages 13 to 15 in FY 2017 to 2018 and eligible for TRICARE benefits in FY 2020 to June 2021 were included in the Behavioral Health cohort. Diagnoses of mood, anxiety, conduct, and attention deficit disorder/attention deficit hyperactivity disorder (ADD/ADHD) were identified using ICD-10 codes [7]. Further, we included suicide ideation (R45.81 or R45.851) and suicide attempt (T14.91 or Z91.5) in our evaluation. Adolescents with concurrent disorders in either time periods were not removed from analysis.

### Analyses

Descriptive statistics were used for patient demographics collected at the time of the health care encounter in FY 2017 to 2018. Patient demographics included gender, age, race or the imputed sponsor’s race, sponsor’s service, and rank, which is a proxy for socio-economic status, and the state of residence which was categorized into U.S. Census Divisions. Due to race only being mandatorily reported for active-duty personnel, a high rate (80% or more) of missing race exists in MHS data. To resolve this issue in our cohort, we imputed missing race with the known race of the adolescent’s sponsor. Percent changes and the Stuart-Maxwell test for marginal homogeneity with a priori of α < 0.05 were performed to assess changes in the cohort BMI categories. To evaluate changes in diagnoses of behavioral health disorders in the cohort, prevalence comparisons as well as percent changes were performed. All analyses were performed using SAS 9.4. This research was conducted under the Center for Health Services Research at the Uniformed Services University of the Health Sciences (USU CHSR) and was found exempt by the university’s Institutional Review Board.

## Results

We identified a total cohort of 160,037 dependent adolescents of the ages 13 to 15 years in FY 2017 to 2018 who were also eligible for TRICARE benefits during the follow-up period of FY 2020 to June 2021. Distribution of cohort demographics from FY 2017 to 2018 can be found in Table 1. The largest demographic groups among adolescents were White race (64.9%), beneficiaries of the Army (42.2%), and with a sponsor in a Senior Enlisted rank (71.5%); age was evenly distributed at approximately 33% each age year.

**Table 1:** Adolescent cohort demographics in FY 2017 to 2018, *N* = 160,037

	**Total Eligible Population (*N* = 160,037)**	
	**Frequency**	**Percent of N**
**Gender**		
Female	81468	50.9
Male	78569	49.1
**Age**		
13	52903	33.9
14	52926	33.1
15	54208	33.9
**Child or Sponsor's Race**		
White	103916	64.9
Black	33999	21.2
Asian/Pacific Islander	12382	7.7
American Indian/Alaska Native	1779	1.1
Other	6820	4.3
Missing	1141	0.7
**Sponsor's Service**		
Army	67577	42.2
Air Force	42791	26.7
Navy	35906	22.4
Marine Corps	13763	8.6
**Sponsor's Rank**		
Junior Enlisted	4594	2.9
Senior Enlisted	114456	71.5
Junior Officer	16880	10.6
Senior Officer	17845	11.2
Warrant Officer	5460	3.4
Other	800	0.5
Missing	< 11	< 11

**Fig. 1:**
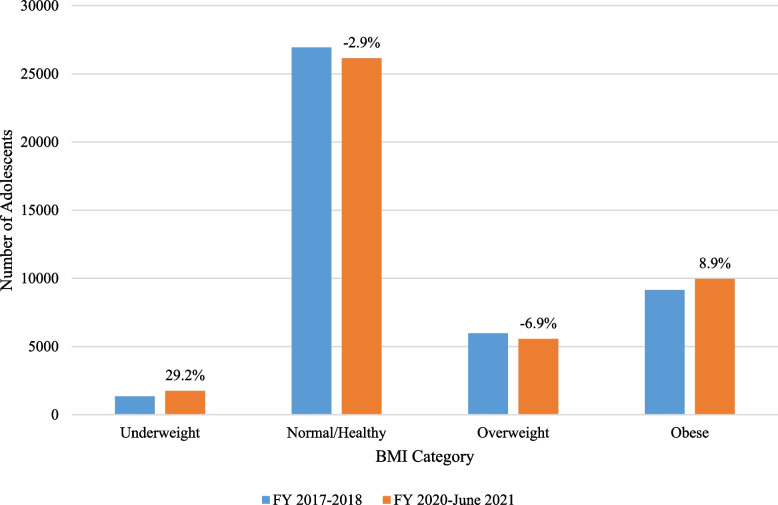
BMI Category comparisons with percent change in adolescent cohort, *n* = 43,394. BMI = Body Mass Index. FY = Fiscal Year

A total of 43,394 dependent adolescents ages 13 to 15 years were included in the BMI cohort. Figure 1 illustrates the comparison of each BMI category prevalence and percent change results. Increases in prevalence and percent change were observed in adolescents with Underweight (29.2%) and Obesity (8.9%), while decreases were observed in adolescents with Healthy Weight (-2.9%) and Overweight (-6.9%).

Due to the increases in adolescents with Underweight and Obesity, we further examined the patient demographics in these categories; the results can be found in Supplemental Materials. In both categories, the majority of adolescents were male, White, beneficiaries of the Army, sponsor with a Senior Enlisted rank, and residing in the South Atlantic division (Supplemental Table 1). In terms of age distribution, adolescents with Underweight were evenly distributed between the starting ages of 13 to 15, while a decrease in distribution from starting age of 13 years to 15 years was observed in adolescents with obesity (Supplemental Table 1).

Additionally, we used percent change analysis to examine shifts in Underweight and Obesity by race). Racial comparison of adolescents with an Underweight BMI found those of Black and ‘Other’ race had the greatest change (51.1%), White (26.6%), and Asian/Pacific Islander (24.7%); and relatively no change was observed in American Indian/Alaskan Native (0.0%) (Supplemental Fig. 1). Racial comparisons of adolescents with Obesity revealed the greatest change in American Indian/Alaskan Native (20.3%), followed by Asian/Pacific Islander (12.0%), White (11.2%), and Black (4.9%), and ‘Other’ race with the least change (2.1%) (Supplemental Fig. 2).

Results from the Stuart-Maxwell test for marginal homogeneity found statistically significant differences (*p* < 0.0001) in all BMI categories from the two time points of measurement (Table 2). The largest shifts were observed in adolescents with Underweight and Overweight in FY 2017 to 2018. Of adolescents with Underweight in the starting period, 44.3% were Healthy Weight in FY 2020 to June 2021. Of adolescents with an Overweight BMI in the starting period, 35.0% were observed with Healthy Weight and 28.5% with Obesity in FY 2020 to June 2021.

**Table 2:** BMI category changes in adolescent cohort Aged 13–15 Years in FY 2017–2018 (*n* = 43,394)^a^

BMI Category in FY 2017–2018	BMI Category in FY 2020-June 2021			
	**Underweight (*n* = 1,740)**	**Healthy Weight (*n* = 26,144)**	**Overweight (*n* = 5,554)**	**Obese (*n* = 9,956)**
	**n (row %)**			
**Underweight (*n* = 1,347)**	704 (52.3)	597 (44.3)	20 (1.5)	26 (1.9)
**Healthy Weight (*n* = 26,935)**	997 (3.7)	22634 (84.0)	2216 (8.2)	1088 (4.0)
**Overweight (*n* = 5,966)**	16 (0.3)	2086 (35.0)	2165 (36.3)	1699 (28.5)
**Obese (*n* = 9,146)**	23 (0.3)	827 (9.1)	1153 (12.6)	7143 (78.1)

^a^Stuart-Maxwell Test for Marginal Homogeneity

*p* < 0.0001

**Fig. 2:**
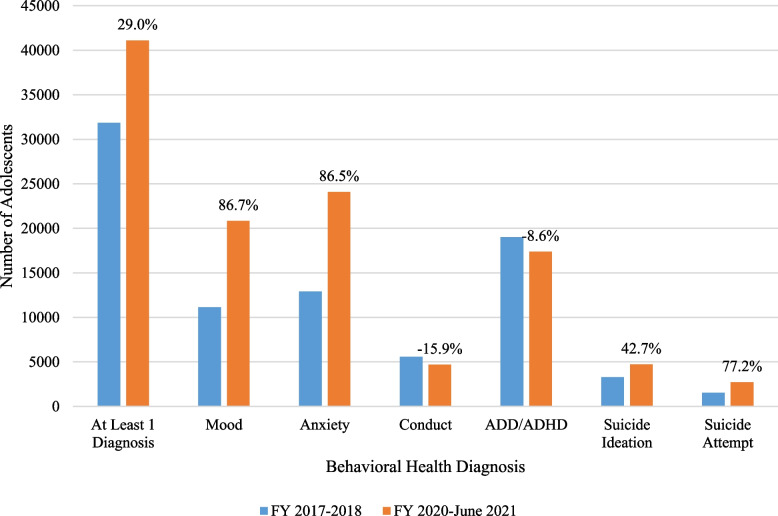
Comparison of behavioral health diagnoses with percent change in adolescent cohort, *n* = 160,037. ADD = Attention Deficit Disorder. ADHD = Attention Deficit Hyperactivity Disorder. FY = Fiscal Year

Total prevalence of and percent change in behavioral health disorders among the total cohort (*N* = 160,037) were evaluated in each period, the results can be found in Fig. 2. Of the total cohort, 19.9% had at least one diagnosed behavioral health disorder in FY 2017 to 2018 and an increase was observed in the follow-up period, with a prevalence of 25.7% and a 29.0% percent change. Upon evaluation of each disorder category, increases in percent change were observed in mood (86.8%) and anxiety (86.5%) disorders, and in suicide ideations (42.7%) and attempts (77.2%). Decreases in conduct disorders (-15.9%) and ADD/ADHD (-8.6%) were also observed.

We further examined these shifts by adolescent race and found the same trends in the total cohort were observed across all races; the results can be found in Supplemental Materials. Adolescents of ‘Other’ race had the highest increases in percent change in having at least one MH diagnosis, mood, suicide ideation, and suicide attempt (Supplemental Table 2). Black adolescents had the greatest increase in anxiety (104.1%), and the greatest decreases in conduct (-20.1%) and ADHD (-19.4%).

## Discussion

### Major findings:

We identified more than 160,037 adolescents from military families, ages 13 to 15 years in FY 2017–2018, who were followed during FY 2020 to June 2021. The cohort was assessed pre-pandemic for body composition (obese, overweight, normal weight, or underweight) and for behavioral diagnoses (mood disorders, anxiety disorders, conduct disorders, and ADD/ADHD), at which time, rates of these conditions were similar to those in the greater U.S. population of adolescents [6] As described in the Introduction, however, children of military families have three potential advantages over their peers: they are universally insured; their military parents have guaranteed employment through the end of their contracts; and they tend to choose military service for themselves at higher rates than do adolescents in the greater U.S. population, raising the possibility of mental and physical fitness as cultural or family expectations. Therefore, the follow-up of this cohort during the height of the pandemic aimed to determine whether military service of at least one parent was protective against increases in overweight, obesity, and behavioral health disorders.

Overall, a majority (> 50%) of adolescents in the Underweight, Healthy Weight, and Obese categories in 2017–2018 remained in those same categories during the follow-up period. Of those in the Overweight category, the largest proportion (36.3%) remained in that category, with approximately 35% decreasing to Healthy Weight. Of the adolescents who were overweight in 2017–2018, 28.5% progressed to obesity in the follow-up period, and a small number (0.27%) progressed to underweight. Of adolescents who were healthy weight in 2017–2018, 4.0% progressed to obesity, 3.7% progressed to underweight, and 8.2% to overweight during the follow-up period. This appears to match data in the general population which documents a rise in child and adolescent obesity, and in the rate of BMI increases, during the COVID-19 pandemic [11, 12]. The Centers for Disease Control and Prevention (CDC) acknowledges that pandemic-related school closures likely reduced children’s access to “structured physical activity” and to healthy foods [12]. Nutritional access could influence both overweight, which is robustly studied in literature, and underweight, which is far less studied in COVID-19 despite being of concern. A 2020 survey by Sharma, et al. [13] revealed food insecurity among 93.5% of respondents during the first year of the pandemic; however, comparative literature associates this more strongly with overweight and obesity than with underweight [14]. The overall rise in BMI among adolescents in military families also mirrors that of the active duty service members themselves, with the Army, Navy, Air Force, and Marine Corps all reporting increases in BMI during the COVID-19 pandemic [15]. Notably, the DoD also moved to a largely remote work platform during this time, with approximately 88% of military and federal civilian respondents reporting a full or partial transition to telework [16]. Taken together, this suggests that neither universal insurance nor military culture was sufficient to eliminate adverse changes in BMI among adolescents or their military parents during the COVID-19 pandemic.

The results for behavioral health were similarly mixed. Prevalence of conduct disorders and ADD/ADHD decreased during the follow-up period; however, prevalence of mood and anxiety disorders, and suicide ideation and suicide attempt, increased during the follow-up period. Conduct disorders and ADD/ADHD co-occur frequently [17], as do anxiety and mood disorders, and therefore each pair would be expected to trend in the same direction. It may be that these pairs are oppositely affected by school closures, with conduct disorders and ADD/ADHD responding positively to a more constrained and predictable home environment, while anxiety and mood disorders responded negatively to the reduced opportunity for social interaction [18]. Alternately, the decrease could be due to reduced access to psychological and primary care during the pandemic, with ADD/ADHD diagnoses especially subject to bias as the manifestations are subtler than for conduct disorders. Effects of the pandemic, access to care, and particularly school closures on youth with conduct disorder and ADD/ADHD are understudied in literature and therefore worthy of future research.

The most concerning findings were the increase in suicide ideation and attempts among adolescents and young adults in this study. The CDC reports an approximately twofold increase in emergency department (ED) visits for suspected suicide attempts in adolescents aged 12–17 during the period of Spring 2020 to Winter 2021, particularly among females [19]. Among young adults aged 18–24, inclusive of the top age range in our study, the CDC reports a decrease in number, but slight (1.1–1.4-fold) increase in rates of ED visits for suspected suicide attempts. The 77% increase in suicide attempts seen in our study falls within the 1.1–twofold increase reported by the CDC. However, our study reports a 43% increase in suicide ideation, in contrast to CDC-reported figures of 25.5% of adolescents aged 18–24 having considered suicide within the last 30 days [20]. This difference may be due to multiple factors including differing lengths of study period, the use of self-reported surveys in the CDC study and the smaller sample size (5470). Further research is needed to address any other factors which may be driving suicide ideation among military adolescents.

While pandemic-associated behavioral health effects are understudied in the active duty population, early work compares the stress of the pandemic to that associated with humanitarian disaster missions, and anticipates similar effects on mental health [21]. The same article also recognizes the common stressors associated with pandemic-related social distancing, which affected military and civilian populations alike [21]. While universal insurance may enable greater access to behavioral health resources, neither universal insurance nor military culture was sufficient to eliminate adverse changes in mental health status among adolescents or their military parents during the COVID-19 pandemic.

### Racial disparities

As described in Results, the largest percentages of adolescents who were underweight (61.8%) or obese (49.4%) during the follow-up period were of White race, which is expected given that White race accounted for 64% of study population **(**Table 1). Black and Other race adolescents had the greatest percent changes (51.1%) in underweight BMI and Native American/ Alaska Native adolescents had the greatest percent change (20.3%) in obesity, compared to the previous study period. The percent change in underweight among Black adolescents is almost double that for White adolescents (26.6% change) and is suggestive of disparity. These issues could include reduced access to food, especially if the non-military parent lost income due to the pandemic; increased physical activity; or eating disorders. These latter, particularly bulimia, may occur in Black adolescent women at a rate 50% higher than in White adolescent women, often as a maladaptive response to stress, while disordered eating is approximately 1/3 as likely to be diagnosed in Black vs. White adolescent women with the same symptoms [22]. Discussion of BMI in U.S. adolescents focuses strongly on overweight and obesity rather than underweight, and comparisons across racial groups are rare. Therefore, these findings represent an important contribution to the literature.

Differences between racial groups were also observed in behavioral health. Black adolescents had notably greater percent increases in anxiety disorders (104.1%) and suicide attempt (90.0%), compared to White adolescents (83.8%, 72.9% respectively). The increase in suicide attempt is particularly concerning, as suicide in 2018 became the third leading cause of death among Black teens aged 15–19 [23]. The National Institutes of Mental Health identifies several factors including differential access to care, and mistrust or negative perception of providers leading to lower completion rates for depression treatment programs, as potential contributors to increased suicide rates in Black adolescents. In our study, this population also showed greater negative percent changes in diagnoses of conduct disorders (-20.1%) and ADHD (-19.4%) compared to White adolescents (-14.7% and -6.1%, respectively). Prior research shows that Black adolescents in the MHS are more likely to be diagnosed with conduct vs. mood disorders, compared to White adolescents with similar symptoms [24]. It is possible that the decrease in conduct disorders reflects a decrease in triggering symptoms outside of a home environment as described earlier, or a decrease in school-based referrals for symptoms which are viewed as problem behaviors. Further research is needed to answer these questions.

Racial differences were also noted for Asian/Pacific Islander adolescents, who had a higher percent increase in anxiety, suicide ideation, and suicide attempt; lower percent increase in mood disorders; and greater percent decrease in ADD/ADHD and conduct disorders, compared to White adolescents. Comparison to published literature shows mixed results, with a 2007 study showing reduced rates of conduct disorders among Asian young adults but increased rates among Native Hawaiian/Pacific Islander young adults, compared to their White counterparts [25], while a 2018 study shows a greater prevalence of anxiety disorders among second but not first-generation Asian young adults [26]. Both prior studies indicate cultural differences as drivers of mental health and mental health care, but these factors could not be assessed from our dataset. The percent increases in suicide ideation (51.6%) and suicide attempt (76.1%) are particularly concerning in Asian and Pacific Islander young adults, as the CDC reports suicide as the second most common cause of death in this population as of 2020 [27]. Further research is needed to identify the risk factors for suicide ideation and attempt in this population and to determine effective, culturally-sensitive interventions.

### Cultural implications of military service

In 2018, approximately 1% of U.S. adults were expected ever to serve in the military [28], and approximately 25% of new recruits are drawn from military families [7]. This significant overrepresentation demonstrates the cultural expectation of military service for young adults in military families, and indicates that this expectation should be considered when designing interventions.

Over 9000 young adults, or approximately 23% of the cohort, were categorized during the follow-up period as obese, which is a barrier to enlistment. An additional 13% were overweight, which is not necessarily a barrier depending on exact BMI and the standards of the Service into which the person is enlisting. For instance, the Army standards for a male recruit of 5 feet, 10 inches tall include a maximum weight of 189 for those 17–20 years of age and 192 for those 21–27 years of age [29], corresponding to an overweight BMI of 27.1 and 27.5 respectively. However, weights above those values would be disqualifying for enlistment even though the BMIs are below the point of obesity. Lifestyle interventions remain the treatment of choice for reducing weight in adolescents, though drugs such as liraglutide and semaglutide are gaining acceptance for those with obesity [30, 31]. Bariatric surgery may be recommended for those with severe obesity [30, 32], but long-term effects on adolescents are not known [30] and the surgery itself is disqualifying for enlistment [8].

Many mental health issues including depression and anxiety are also disqualifying, if care was received for 12 cumulative months or within 36 months prior to accession [33]. Our data shows 23% of young adults having at least one mental health or behavioral health diagnosis during the follow-up period, representing nearly 88,000 people ineligible for service. Many military programs focus on family-level strategies, particularly for those with school-aged children [34, 35], which may serve a protective role against poor mental and behavioral health and thereby preserve fitness to serve. However, while some of the programs offered may be evidence-based, the most widely available are not externally validated and may not may not meet the needs of young adults vs. school-age children. Further research is needed to address this issue.

### Next steps and recommendations

As described in our previous work, children in military families have access to a number of resources designed to preserve mental and physical fitness, including both school-based and military-provided programs. However, schools and universities were closed or operating on virtual status during much of 2020 and early 2021 due to the COVID pandemic, limiting access to many programs. This is particularly important given the increased rate of suicide attempts seen in our cohort. The Department of Defense (DoD) should invest in a multi-layered health infrastructure as recommended by the CDC [19], and should ensure sure that interventions are easily accessible even during times of disruption, are evidence based, and are validated. Further research should also be performed to determine whether those at risk for suicide are able to access care through the MHS and whether they are alternatively seeking care outside the MHS. Similarly, the DoD should also prepare to implement validated health and fitness interventions where and when these are not accessible through the schools or communities.

### Limitations

As with all studies that rely on claims data, this one is subject to potential errors in coding, such as misclassification and underreporting, and the loss of clinical nuance as notes were not captured in this data set. Of the total cohort, we were only able to identify 11% with a BMI recorded in both time periods, which could potentially lead to significantly underestimating the changes observed in MHS adolescents; however, our data reflects similar findings observed in national statistics during the pandemic. In-person healthcare services were significantly reduced during the pandemic as well, which could have impacted our behavioral health prevalence and BMI estimates. As noted above, combination of demographic categories such as Asian with Pacific Islander, and small numbers in some populations such as Native American/Alaska Native, hamper full analysis of factors affecting mental and behavioral health. This study does not clearly separate the effects of age from effects of the pandemic on the rates of behavioral health diagnoses; for instance, in ADD/ADHD which is commonly diagnosed in children and for which rates are reasonably expected to decline during adolescence, or for anxiety and depression which are known to increase during the teen years. Although findings in the greater U.S. population support the idea of the pandemic having negative effects on adolescent behavioral health, further research would be needed to separate conclusively the effects of progression through adolescence from the effects of the pandemic. Finally, this study does not capture care paid for outside of the MHS, such as peer or spiritual counseling for suicide ideation. This is particularly important to consider in light of fitness to serve, for which thoughts of and attempts at suicide are considered disqualifying conditions [9].

## Conclusions

During the first year of the COVID-19 pandemic, adolescents in military families experienced significant changes to physical and mental health status, comparable to the effects in non-military families, indicating that universal insurance, generally guaranteed parental employment, and parental focus on physical and mental fitness standards were not protective. Progression to underweight may be driven by eating disorders not captured in this study or to reduced access to healthy foods. Progression to obesity was likely driven by reduced access to healthy food, increase in convenience food, and a sedentary lifestyle during periods of shutdown. Approximately 88,000 young adults were diagnosed with at least one behavioral condition during the study period. The increases in suicide ideation and attempt are particularly concerning, as are the greater percent increases for minority-race vs. White adolescents. The Department of Defense should invest in multilayered, evidence-based, validated resources for promotion of physical and mental health among adolescent beneficiaries, particularly in times when school and community resources are unavailable, and with specific focus on those at risk for suicidality or suicide ideation. Providers should prepare to consider the expectation of military service as a cultural factor to be accommodated, and where possible and appropriate, design interventions that preserve these young adults’ fitness to serve.

### Supplementary Information

**Additional file 1: Supplemental Figure 1. **Prevalence and Percent Change in Underweight BMI by Race.

**Additional file 2: Supplemental Figure 2. **Prevalence and Percent Change in Obesity by Race.

**Additional file 3: Table S1. **Demographics of Adolescents by Underweight and Obese BMI Recorded in FY 2020 to June 2021.

**Additional file 4: Table S2.** Percent Change in the Number of Adolescents with a Behavioral Health Diagnoses by Race.

## Data Availability

The data that support the findings of this study are available from the Department of Defense via the Defense Health Agency, but restrictions apply to the availability of these data, which were used under license for the current study, and so are not publicly available. Data are however available from Dr. Tracey Koehlmoos, upon reasonable request and with permission of the Defense Health Agency.

## Abbreviations

ADD: Attention Deficit Disorder
ADHD: Attention Deficit/Hyperactivity Disorder
BMI: Body Mass Index
CDC: Centers for Disease Control and Prevention
COVID-19: Coronavirus Disease 2019
DC: Direct Care
DoD: Department of Defense
FY: Fiscal Year
ICD-10: International Classification of Diseases, 10^th^ Edition
MDR: Military Health System Data Repository
MHS: Military Health System
PSC: Private Sector Care
U.S: United States
