# Supplementary material for: Changes in body mass index and behavioral health among adolescents in military families during the COVID-19 pandemic: a retrospective cohort study
Source: BMC Public Health. 2023 Aug 24;23:1615. doi: 10.1186/s12889-023-16548-0 (PMC10463909; doi:10.1186/s12889-023-16548-0)
Supplement: Supplementary file 1 — Additional file 1: Supplemental Figure 1. Prevalence and Percent Change in Underweight BMI by Race. [file 12889_2023_16548_MOESM1_ESM.docx]

**Supplemental Figure 1. Prevalence and Percent Change in Underweight BMI by Race**

BMI= Body Mass Index. FY= Fiscal Year
