# Supplementary material for: Changes in body mass index and behavioral health among adolescents in military families during the COVID-19 pandemic: a retrospective cohort study
Source: BMC Public Health. 2023 Aug 24;23:1615. doi: 10.1186/s12889-023-16548-0 (PMC10463909; doi:10.1186/s12889-023-16548-0)
Supplement: Supplementary file 2 — Additional file 2: Supplemental Figure 2. Prevalence and Percent Change in Obesity by Race. [file 12889_2023_16548_MOESM2_ESM.docx]

**Supplemental Figure 2. Prevalence and Percent Change in Obesity by Race**

FY= Fiscal Year
