# Supplementary material for: Changes in body mass index and behavioral health among adolescents in military families during the COVID-19 pandemic: a retrospective cohort study
Source: BMC Public Health. 2023 Aug 24;23:1615. doi: 10.1186/s12889-023-16548-0 (PMC10463909; doi:10.1186/s12889-023-16548-0)
Supplement: Supplementary file 3 — Additional file 3: Table S1. Demographics of Adolescents by Underweight and Obese BMI Recorded in FY 2020 to June 2021. [file 12889_2023_16548_MOESM3_ESM.docx]

**Table S1. Demographics of Adolescents by Underweight and Obese BMI Recorded in FY 2020 to June 2021**

|  | **Underweight (n=1,740)** | **Obese (n=9,960)** |
| --- | --- | --- |
|  | **n (column %)** | |
| **Patient Sex** |  |  |
| Female | 650 (37.4) | 4734 (47.5) |
| Male | 1090 (62.6) | 5226 (52.6) |
| **Patient Age (in FY 2017-2018)** |  |  |
| 13 | 550 (31.6) | 3731 (37.5) |
| 14 | 599 (34.4) | 3308 (33.2) |
| 15 | 591 (34.0) | 2921 (29.3) |
| **Race** |  |  |
| White | 1076 (61.8) | 4918 (49.4) |
| Black | 278 (16.0) | 2354 (23.6) |
| Asian/Pacific Islander | 111 (6.4) | 702 (7.1) |
| American Indian/Alaskan Native | <11 | 77 (0.8) |
| Other | 71 (4.1) | 393 (4.0) |
| Missing | 195 (11.2) | 1516 (15.2) |
| **Sponsor's Branch of Service** |  |  |
| Army | 666 (38.3) | 4567 (45.9) |
| Air Force | 570 (32.8) | 2434 (24.4) |
| Navy | 391 (22.5) | 2236 (22.5) |
| Marine Corps | 113 (6.5) | 723 (7.3) |
| **Sponsor's Rank** |  |  |
| Junior Enlisted | 39 (2.2) | 307 (3.1) |
| Senior Enlisted | 1118 (64.3) | 6731 (67.6) |
| Junior Officer | 131 (7.5) | 629 (6.3) |
| Senior Officer | 219 (12.6) | 649 (6.5) |
| Warrant Officer | 48 (2.8) | 210 (2.1) |
| Other | 183 (10.5) | 1421 (14.3) |
| Missing | <11 | 13 (0.1) |

BMI= Body Mass Index
