# Supplementary material for: Changes in body mass index and behavioral health among adolescents in military families during the COVID-19 pandemic: a retrospective cohort study
Source: BMC Public Health. 2023 Aug 24;23:1615. doi: 10.1186/s12889-023-16548-0 (PMC10463909; doi:10.1186/s12889-023-16548-0)
Supplement: Supplementary file 4 — Additional file 4: Table S2. Percent Change in the Number of Adolescents with a Behavioral Health Diagnoses by Race. [file 12889_2023_16548_MOESM4_ESM.docx]

**Table S2. Percent Change in the Number of Adolescents with a Behavioral Health Diagnoses by Race**

| **Behavioral Health Diagnosis** | **White** | **Black** | **Asian/Pacific Islander** | **American Indian/Alaskan Native** | **Other** |
| --- | --- | --- | --- | --- | --- |
|  | **Percent Change from FY 17-18 to FY 2020-June 21** | | | | |
| At least one diagnosis | 29.9% | 23.7% | 32.1% | 25.1% | 33.9% |
| Mood | 85.9% | 89.8% | 78.2% | 94.0% | 102.7% |
| Anxiety | 83.8% | 104.1% | 86.3% | 82.1% | 88.5% |
| Conduct | -14.7% | -20.1% | -19.9% | -18.6% | -9.8% |
| ADD/ADHD | -6.1% | -19.4% | -8.0% | -11.4% | -5.3% |
| Suicide Ideation | 41.3% | 41.4% | 51.6% | 31.1% | 73.0% |
| Suicide Attempt | 72.9% | 90.0% | 76.1% | 52.0% | 131.5% |

ADD/ADHD= Attention Deficit Disorder/Attention Deficit Hyperactivity Disorder; FY=Fiscal Year
